# The Homunculus of unspecific bone uptakes associated with PSMA-targeted tracers: a systematic review-based definition

**DOI:** 10.1007/s00259-024-06797-5

**Published:** 2024-06-17

**Authors:** Alessio Rizzo, Silvia Morbelli, Domenico Albano, Giuseppe Fornarini, Martina Cioffi, Riccardo Laudicella, Francesco Dondi, Serena Grimaldi, Francesco Bertagna, Manuela Racca, Giorgio Treglia, Matteo Bauckneht

**Affiliations:** 1 Nuclear Medicine, Candiolo Cancer Institute, FPO–IRCCS, Turin, Italy; 2 Nuclear Medicine, AOU Città Della Salute e Della Scienza di Torino, Turin, Italy; 3 University of Turin, Turin, Italy; 4 Nuclear Medicine, ASST Spedali Civili of Brescia, Brescia, Italy; 5 Radiological Sciences and Public Health Department, University of Brescia, Brescia, Italy; 6 IRCCS Ospedale Policlinico San Martino, Genova, Italy; 7 Nuclear Medicine, Department of Biomedical and Dental Sciences and Morpho-Functional Imaging, University of Messina, Messina, Italy; 8 Nuclear Medicine, Imaging Institute of Southern Switzerland, Ente Ospedaliero Cantonale, Bellinzona, Switzerland; 9 Faculty of Biology and Medicine, University of Lausanne, Lausanne, Switzerland; 10 Faculty of Biomedical Sciences, Università della Svizzera Italiana, Lugano, Switzerland; 11 Nuclear Medicine, Department of Health Sciences (DISSAL), University of Genova, Genova, Italy

**Keywords:** Bone metastastes, PET, Prostate cancer, Prostate specific membrane Antigen, Positron Emission Tomography, UBU

## Abstract

**Purpose:**

Prostate-Specific Membrane Antigen (PSMA)-targeted Positron Emission Tomography (PET) has revolutionised prostate cancer (PCa) diagnosis and treatment, offering superior diagnostic accuracy over traditional methods and enabling theragnostic applications. However, a significant diagnostic challenge has emerged with identifying unspecific bone uptakes (UBUs), which could lead to over-staging and inappropriate treatment decisions if misinterpreted. This systematic review explores the phenomenon of UBUs in PCa patients undergoing PSMA-PET imaging.

**Methods:**

Studies assessing the prevalence, topographical distribution, and potential clinical implications of UBUs were selected according to the Preferred Reporting Items for a Systematic Review and Meta-Analysis (PRISMA) method and evaluated with the Quality Assessment of Diagnostic Accuracy Studies (QUADAS-2) tool.

**Results:**

The percentage of PCa patients with UBUs on PSMA-PET scans ranged from 0 to 71.7%, depending on the radiopharmaceutical used, with [^18^F]PSMA-1007 showing the highest incidence. The ribs are the primary site of UBUs across all PSMA-targeted radiopharmaceuticals. The spine is the second most frequent UBU site for [^68^Ga]Ga-PSMA-11, [^18^F]DCFPyL, [^18^F]rhPSMA-7, while the pelvic girdle represents the second most frequent site for [^18^F]PSMA-1007. The average maximum Standardized Uptake Value (SUV_max_) of UBUs varied from 3.4 to 7.7 and was generally lower than that of bone metastases.

**Conclusions:**

Our findings underscore the need for heightened awareness and precise interpretation of UBUs to avoid potential over-staging and subsequent inappropriate treatment decisions. Considering the radiopharmaceutical used, PET-derived semiquantitative parameters, the topographical distribution of UBUs, and accurately evaluating the pre-test probability based on clinical and laboratory parameters may aid nuclear medicine physicians in interpreting PSMA-PET findings.

**Supplementary Information:**

The online version contains supplementary material available at 10.1007/s00259-024-06797-5.

## Introduction

Recent advances in prostate cancer (PCa) management have been significantly influenced by the advantages of PSMA-targeted PET scans over traditional diagnostics, paving the way for their use as theragnostic agents [1]. Despite initial treatments like radiation or surgery, up to 60% of PCa patients can face biochemical recurrence (BCR) within a decade. Early identification of disease sites enables targeted interventions such as local salvage therapy for relapses or metastatic ablation for oligometastatic PCa, providing possible curative alternatives to palliative androgen-deprivation therapy [2, 3].

Considering the high expression of PSMA on the cell membrane of PCa cells and based on the first urea-based compounds, several low-molecular-weight radiolabelled PSMA inhibitors have been developed to expand the diagnostic performance of nuclear medicine imaging for PCa detection. Currently, the most commonly used PSMA-targeting radiopharmaceutical worldwide is [^68^Ga]Ga-PSMA-11, also known as [^68^Ga]Ga-DKFZ-PSMA-11 or [^68^Ga]Ga-PSMA-HBED-CC [4–7]. Recently, [^18^F]labeled PSMA-targeting radiopharmaceuticals were widely adopted into clinical practice, mainly with [^18^F]DCFPyL and [^18^F]PSMA-1007 [8, 9]. Unlike other PSMA radioligands, [^18^F]PSMA-1007 has increased lipophilicity and is primarily eliminated by the liver. This characteristic may reduce nonspecific activity in the ureter and bladder, potentially mitigating urinary excretion issues [10]. [^18^F]-labelled options potentially offer reduced costs, broadened availability, and superior image quality due to lower positron energy [11]. However, with the progressive increase in the number of facilities performing PSMA-targeted PET worldwide and the expanding body of literature, a limitation of this relatively novel diagnostic probe is represented by unspecific bone uptakes (UBUs) [12]. If misinterpreted, these false positive findings could result in PCa over-staging and lead to erroneous treatment choices (i.e., palliative over radical therapy).

Several studies have recently investigated the incidence of UBUs in PET imaging using different PSMA-targeting radiopharmaceuticals among PCa patients across various clinical settings. This systematic review aims to collect the available literature on this PSMA-PET potential drawback, highlight the main differences among the most used radiopharmaceuticals, and summarise the topographical quantitative distribution of these findings.

## Materials and methods

### Protocol, review question and inclusion criteria

Based on a preconceived protocol [13], the current systematic review was developed referring to the “Preferred Reporting Items for a Systematic Review and Meta-Analysis” (PRISMA 2020 statement) [14]. The comprehensive PRISMA checklist is available in the Supplementary Table 1. The systematic review has been preregistered on the PROSPERO database (protocol number CRD42024519876).

A review question was defined based on the “Population, Intervention, Comparator, Outcomes” framework (PICO): what is the prevalence of UBUs (outcome) on PSMA-targeted PET imaging (intervention) in patients diagnosed with PCa (patient/population)? The presence of a comparator was not considered an exclusion criterion. Two authors (M.B. and A.R.) independently conducted the literature search, study selection, quality assessment, and data extraction. Disagreements were resolved through an online meeting with a third reviewer (S.M.). Reviews, editorials, comments, case reports, and original investigations on different topics were excluded. No language restriction was applied.

### Literature search strategy, selection process, data collection and extraction

The authors comprehensively searched for articles dealing with UBUs on PSMA-targeted PET images, employing two electronic bibliographic databases (Scopus and PubMed/MEDLINE). The search algorithm included the following terms: (“PSMA” OR “Prostate Specific Membrane Antigen”) AND (“unspecific” OR “not specific” OR “non-specific” OR “nonspecific” OR “indeterminate” OR “undetermined” OR “uncertain” OR “unclear” OR “UBU”) AND (“bone” OR “skelet*”)). Moreover, reviewers screened included studies’ references, searching for additional eligible articles meeting the predetermined inclusion criteria. The literature search was last updated on 25.02.2024.

The reviewers independently read the titles and abstracts of the records generated by the search algorithm. They then determined which studies were eligible based on predefined criteria. Thereafter, the reviewers collected data from all of the included studies, taking advantage of full-text, tables, and supplemental material regarding general study information (authors, publication year, country, study design, funding sources), patients’ characteristics (sample size, age, clinical setting, Gleason score, serum markers levels), PET-related details (administered radiopharmaceuticals and their activity, hybrid imaging protocol, image analysis method), and outcome (including UBU prevalence, UBU sites, average radiopharmaceutical uptake for UBU, and UBU validation method).

### Quality assessment (risk of bias assessment)

QUADAS-2 was used to assess the quality of the included studies, to analyse the risk of bias, and to determine their pertinence to the review question [15]. To perform the quality assessment, the reviewers considered four domains (patient selection, index test, reference standard, flow, and timing). To assess the applicability of the included studies, they considered three categories (patient selection, index test, and reference standard).

### Literature analysis

Due to the heterogeneity of the available studies and the absence of quantitative data in more cases, we planned a systematic review (qualitative synthesis) without a meta-analysis (quantitative synthesis). Therefore, a statistical analysis (pooled analysis) was not performed.

## Results

### Study characteristics

**Fig. 1:**
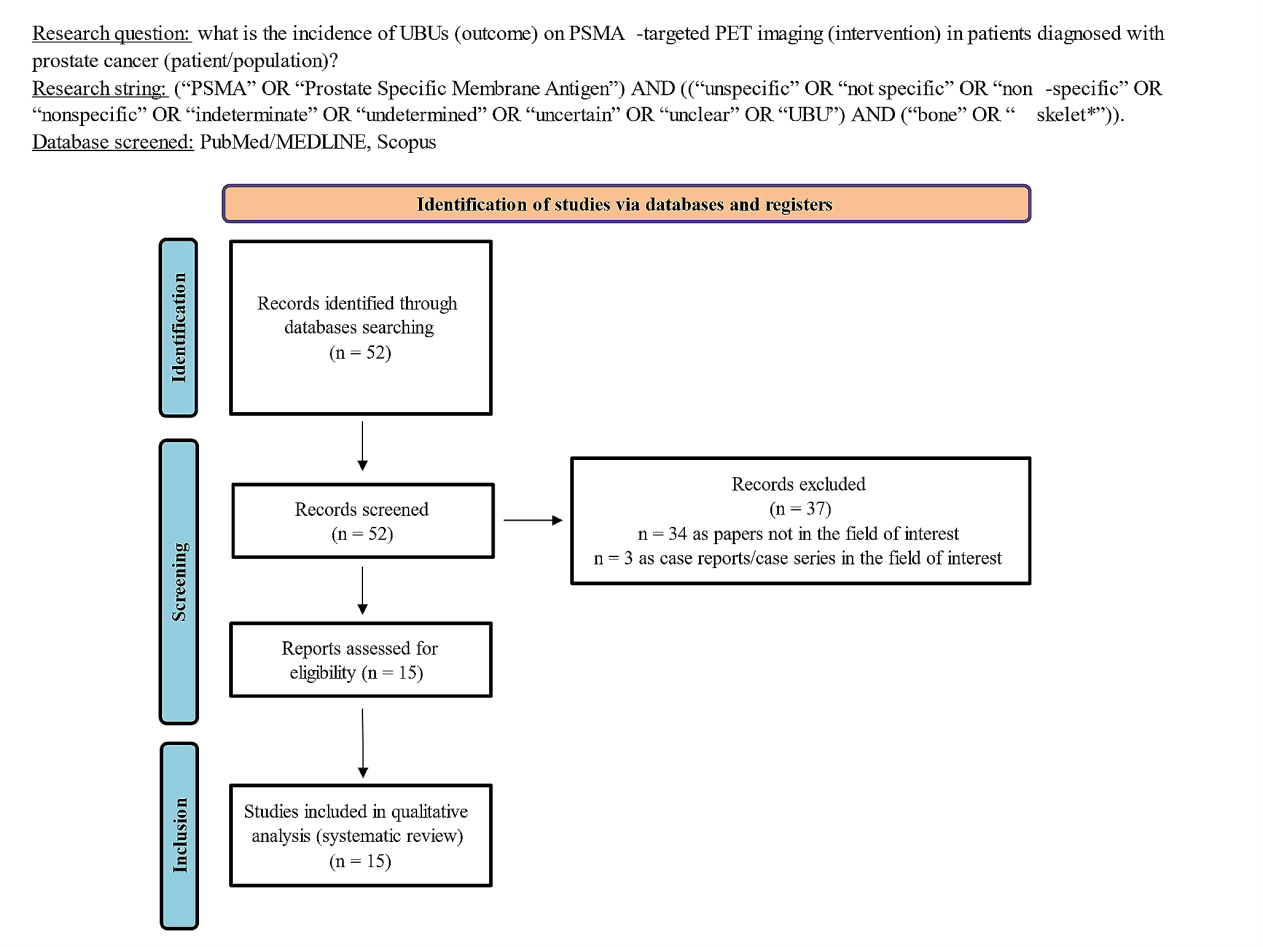
Overview of the study selection process

Fifteen studies satisfied the inclusion criteria [16–30]. The study selection process is summarized in Fig. 1. All the included articles except two accounted for a retrospective design [16–23, 25, 27–30], whereas the remaining trials were prospective [24, 26]. Only two papers reported a multicentric design [22, 27]. Table 1 summarises the general information of the included studies.

**Table 1:** General data of the included studies

Authors [Ref.]	Year	Country	Study design/number of involved centres	Were UBU the primary outcome?	
Chiu et al. [16]	2020	U.S.A.	Retrospective / Single centre	yes	
Chen et al. [17]	2020	Australia	Retrospective / Single centre	yes	
Rauscher et al. [18]	2020	Germany	Retrospective / Single centre	yes	
Dietlein et al. [19]	2020	Germany	Retrospective / Single centre	no	
Arnfield et al. [20]	2021	Australia	Retrospective / Single centre	yes	
Hoberück et al. [21]	2021	Germany	Retrospective / Single centre	no	
Grünig et al. [22]	2021	Switzerland	Retrospective /Multicentric	yes	
Kroenke et al. [23]	2021	Germany	Retrospective / Single centre	no	
Pattinson et al. [24]	2022	Australia	Prospective / Single centre	no	
Vollnberg et al. [25]	2022	Switzerland	Retrospective / Single centre	yes	
Phelps et al. [26]	2022	U.S.A.	Prospective / Single centre	yes	
Letang et al. [27]	2022	France	Retrospective / Multicentric	yes	
Ninatti et al. [28]	2023	Italy	Retrospective / Single centre	yes	
Seifert et al. [29]	2023	Germany	Retrospective / Single centre	yes	
Luo et al. [30]	2024	China	Retrospective / Single centre	yes	

Table 2 presents the clinical characteristics of PCa patients from various studies. The number of participants varied from 10 to 792 (age range: 67-72.1). In four studies, PSMA PET was used for restaging PCa patients [18, 19, 27, 29], while two studies were conducted for primary staging [17, 28]. The remaining nine studies utilised PSMA PET imaging in both contexts [16, 20–26, 30]. When provided, the Gleason score for grading the included patients was reported as International Society of Urological Pathology (ISUP) Grade Group 1 in two patients, ISUP 2 in 576 patients, and ISUP 3 in 399 patients [16, 18, 20, 21, 23–28]. Regarding prostate-specific antigen (PSA) serum levels, the average values reported ranged from 0.8 to 110 ng/mL [19].

**Table 2:** Clinical characteristics of PCa patients from the included studies

Authors [Ref.]	Sample size	Mean/Median age (Years)	Clinical setting (no. patients)	Gleason score (no. patient)	PSA values (ng/mL)
Chiu et al. [16]	56	Mean: 67	Staging: 18 Restaging: 38	6–7: 21 8 − 10: 35	Median: 13.7
Chen et al. [17]	111	Median: 68	Staging: 111	not available	Mean: 10.1
Rauscher et al. [18]	[^18^F]PSMA-1007: 102	Median: 70	Restaging: 102	6–7:63 8 − 10: 39	Median: 0.87
	[^68^Ga]Ga-PSMA-11: 102	Median: 69	Restaging: 102	6–7:63 8 − 10: 39	Median: 0.91
Dietlein et al. [19]	27	Mean: 67.2	Restaging: 27	not available	not available
Arnfield et al. [20]	214	Mean: 69.6	Restaging: 114 Staging: 100	6–7: 107 8-10: 80	Median UBU+: 5.2 Median UBU-: 4.85
Hoberück et al. [21]	46	Median: 71	Restaging: 36 Staging: 10	6–7: 22 8 − 10: 24	Median: 3.8
Grünig et al. [22]	348	Median: 71	Restaging: 227 Tumour evaluation: 71 Staging: 49	not available	Median: 306
Kroenke et al. [23]	[^18^F]rhPSMA-7: 160	Median: 72	Restaging: 127 Staging: 33	6–7: 80 8 − 10: 47	Median: Restaging: 0.9 Staging: 14
	[^68^Ga]Ga-PSMA-11: 160	Median: 69	Restaging: 127 Staging: 33	6–7: 79 8 − 10: 48	Median: Restaging: 2.1 Staging: 10.1
Pattinson et al. [24]	50	Mean: 71.8	Restaging: 27 Metastatic: 11 Staging: 12	6–7: 21 8 − 10: 21	Median Restaging: 0.8 Metastatic: 9.7 Staging: 12
Vollnberg et al. [25]	10	Median: 66	Restaging: 9 Staging: 1	6–7: 8 8 − 10: 2	Mean Restaging: 1.8 Staging: 110
Phelps et al. [26]	243	Median: 66	Restaging: 35 Staging: 13	< 6: 2 6 − 7: 24 8 − 10: 22	Median: 4.0
Letang et al. [27]	53	Median: 71	Restaging: 53	6–7: 46 8 − 10: 7	Mean: 2.9
Ninatti et al. [28]	77	Median: 67	Staging: 77	6–7: 42 8 − 10: 35	Median: 7
Seifert et al. [29]	[^18^F]PSMA-1007: 409	Median: 71	Restaging: 792	not available	< 1: 430 1 < PSA < 5: 285 > 5: 42
	[^68^Ga]Ga-PSMA-11: 383				
Luo et al. [30]	105	Mean: 72.1	Restaging: 37 Staging: 68	not available	Median: 16.2

Technical details of the included studies are reported in Table 3. All included studies qualitatively evaluated UBUs, with thirteen conducting a semiquantitative analysis to extract the standardised uptake values (SUV) [16, 18, 20–30].

**Table 3:** Index test key characteristics

Authors [Ref.]	Tracer	Hybrid imaging	Average administered activity	Uptake time (minutes)	Image analysis
Chiu et al. [16]	[^68^Ga]Ga-PSMA-11	PET/CTPET/MRI	207 MBq	67	Qualitative, Semiquantitative (SUV_max_)
Chen [17]	[^68^Ga]Ga-PSMA-11	PET/CT	not available	not available	Qualitative
Rauscher et al.* [18]	[^18^F]PSMA-1007	PET/CT	325 MBq	94	Qualitative, Semiquantitative (SUV_max_)
	[^68^Ga]Ga-PSMA-11	PET/CT	147 MBq	54	
Dietlein et al.** [19]	[^18^F]PSMA-1007	PET/CT	343 MBq	120	Qualitative
	[^68^Ga]Ga-PSMA-11 [^18^F]DCFPyL [^18^F]-JK-PSMA-7		[^68^Ga]Ga-PSMA-11: 159 MBq [^18^F]DCFPyL: 343 MBq [^18^F]-JK-PSMA-7: 323 Mbq	60	
Arnfield et al. [20]	[^18^F]PSMA-1007	PET/CT	250 MBq	126	Qualitative; Semiquantitative (SUV_max_)
Hoberück et al** [21]	[^18^F]PSMA-1007	PET/CT	154 MBq	103	Qualitative; Semiquantitative (SUV_max,_ SUV_peak,_ SUV_mean_)
	[^68^Ga]Ga-PSMA-11		149.3 MBq	106	
Grünig et al. [22]	[^18^F]PSMA-1007	PET/CT PET/MRI	3–4 MBq/kg	60–90	Qualitative; Semiquantitative (SUV_max_)
Kroenke et al.* [23]	[^18^F]rhPSMA-7: 160	PET/CT	329 Mbq	80	Qualitative; Semiquantitative (SUV_max_)
	[^68^Ga]Ga-PSMA-11: 160	PET/CT	143 MBq	45	Qualitative; Semiquantitative (SUV_max_)
Pattinson et al.** [24]	[^18^F]PSMA-1007	PET/CT	250 MBq	120–180	Qualitative; Semiquantitative (SUV_max_, SUV_mean_)
	[^68^Ga]Ga-PSMA-11		100–150 MBq	45–60	
Vollnberg et al. [25]	[^18^F]PSMA-1007	PET/CT	240 MBq	90	Qualitative; Semiquantitative (SUV_max_)
Phelps et al. [26]	[^18^F]DCFPyl	PET/CT	267	120	Qualitative; Semiquantitative (SUV_max_)
Letang et al. [27]	[^68^Ga]Ga-PSMA-11	PET/CT	2.2 MBq/kg	63	Qualitative; Semiquantitative (SUV_max_)
Ninatti et al. [28]	[^18^F]PSMA-1007	PET/CT PET/MRI	not available	not available	Qualitative
Seifert et al* [29]	[^18^F]PSMA-1007	PET/CT	350.6 MBq	111	Qualitative; Semiquantitative (SUV_max_)
	[^68^Ga]Ga-PSMA-11		133.3 MBq	81	
Luo et al. [30]	[^18^F]PSMA-1007	PET/CT	3.7 MBq/kg	97	Qualitative; Semiquantitative (SUV_max_)

*: the study design involved an interindividual comparison of the administered tracers

**: the study design involved an intraindividual comparison of the administered tracers

Seven studies conducted bone biopsies on at least one patient regarding the reference standard used to assess the aetiology of focal bone uptakes [17, 20, 22, 25–27, 30], while ten studies used a composite reference standard with or without biopsy [16–20, 22, 26, 27, 29, 30]. Four studies lacked methods to verify if bone uptakes were UBUs or misdiagnosed metastases [21, 23, 24, 28].

### Risk of bias and applicability

**Fig. 2:**
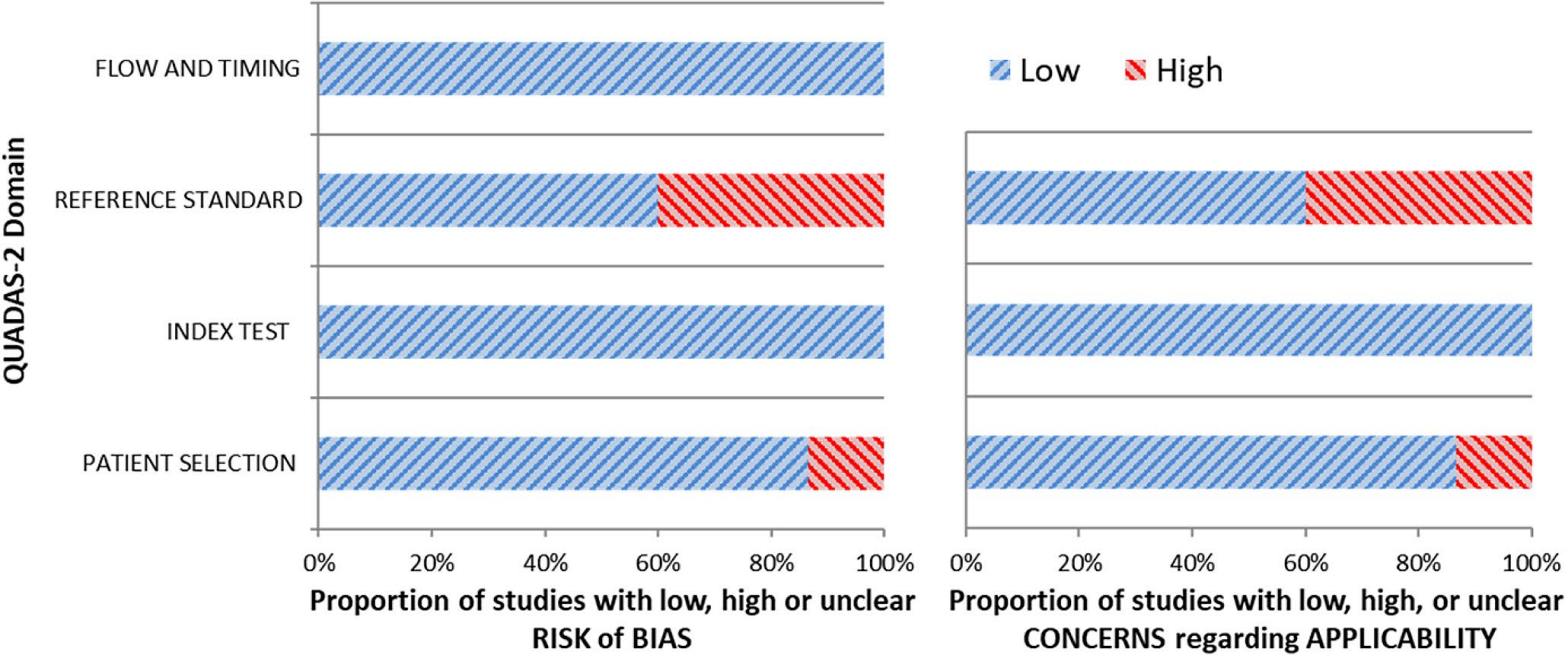
Quality assessment according to QUADAS-2 tool. Authors classified the papers included in the systematic review as high- or low-risk of bias or applicability concerns for distinct domains listed in the ordinate axis. The graph indicates that almost 40% of the included studies are affected by a high risk of bias in the “reference standard” domain

Reviewers used the QUADAS-2 tool to assess the relevance of each paper based on reported data. Figure 2 briefly resumes the concerns about the quality and applicability of the included research.

## Results of individual studies (qualitative synthesis)

When assessed, the percentage of PCa patients with UBUs ranged from 11.6 to 71.7% for [^18^F]PSMA-1007 [18–22, 24, 28–30], from 0 to 23.9% for [^68^Ga]Ga-PSMA-11 [16, 18, 21, 24, 29], and was 19.8% for the single study using [^18^F]DCFPyl [26]. Concerning the semiquantitative metrics, the average UBUs SUV_max_ values varied from 3.4 to 7.7 for [^18^F]PSMA-1007 and 4.6 to 5 for [^68^Ga]Ga-PSMA-11. When reported, UBUs uptake was significantly lower than bone metastases [20, 24, 26, 27, 30]. UBUs incidence and uptake characteristics reported by the selected studies are summarized in Table 4.

**Table 4:** Study outcomes. *: The study enrolled only patients with one or more bone uptakes

Authors [Ref.]	UBU Mean/Median SUV_max_		Number of UBUs (*n* of patients/ %)	Is there a significant uptake difference between UBUs and metastases?	UBU validation method	UBU etiology (when biopsied)	
Chiu et al. [16]	n.a.		13/23%	not available	PSA follow-up	not available	
Chenet al.* [17]	not available		111/100%	not available	Imaging follow-up; biopsy	Benign tissue (Myoblastic proliferation)	
Rauscher et al. [18]	[^18^F]PSMA-1007: 5.5		49/48%	not available	PSA follow-up	not available	
	[^68^Ga]Ga-PSMA-11: 4.6		15/14.7%				
Dietlein et al. [19]	[^18^F]PSMA-1007: 7.74		[^18^F]PSMA-1007: 7/25.9%	not available	Imaging follow-up	not available	
	Renally-excreted PSMA ligands: not available		Renally-excreted PSMA ligands: 0/0%				
Arnfield et al. [20]	Median: 3.4		94/43.9%	Yes	PSA follow-up; clinical follow-up; biopsy	Faint fibroblastic reaction (Fibrous dysplasia)	
Hoberück et al. [21]	not available		[^18^F]PSMA-1007: 33/71.7%	not available	not available	not available	
			[^68^Ga]Ga-PSMA-11: 11/23.9%				
Grünig et al. [22]	4.2 ± 2.0		179/54.4%	not available	PSA follow-up; Imaging follow-up; biopsy	28 unknown origin; 28 benign condition (hyperplastic bone marrow; Paget’s disease); 9 bone metastasis	
Kroenke et al. [23]	[^18^F] rhPSMA-7: 6.1 ± 2.9		Absolute number of UBU: 120	not available	not available	not available	
	[^68^Ga]Ga-PSMA-11: 5 ± 2.4		Absolute number of UBU: 56				
Pattinson et al. [24]	[^18^F]PSMA-1007: 6.2		6/12%:	Yes	not available	not available	
	[^68^Ga]Ga-PSMA-11: 2.4		0/0%				
Vollnberg et al.* [25]	Mean: 18.8 ± 13.1		12/100%	not available	Biopsy	10 benign condition (unknown origin); 1 bone metastasis	
Phelps et al. [26]	Median: 3.6		48/19.8%	Yes	PSA follow-up; imaging follow-up; biopsy	2 bone marrow fibrous replacement; 3 physiologic bone marrow; 3 bone metastasis	
Letang et al.* [27]	Mean: 7.2 ± 7.6		53/100%	Yes	Imaging follow-up; biopsy	not available	
Ninatti et al. [28]	not available		29/37.7%	not available	not available	not available	
Seifert et al. [29]	not available		[^18^F]PSMA-1007: 140/34.2%	not available	Imaging follow-up	not available	
			[^68^Ga]Ga-PSMA-11: 64/16.7%				
Luo et al. [30]	Median: 4.7		169 / 11.6%	Yes	Imaging follow-up; biopsy	not available	

One study assessed the differences in the incidence of UBUs by comparing PET scans performed in different centres and observed a significantly higher number of UBUs in digital than analogue PET [22]. The same paper correlated the uptake time with the incidence of UBUs [22].

**Fig. 3:**
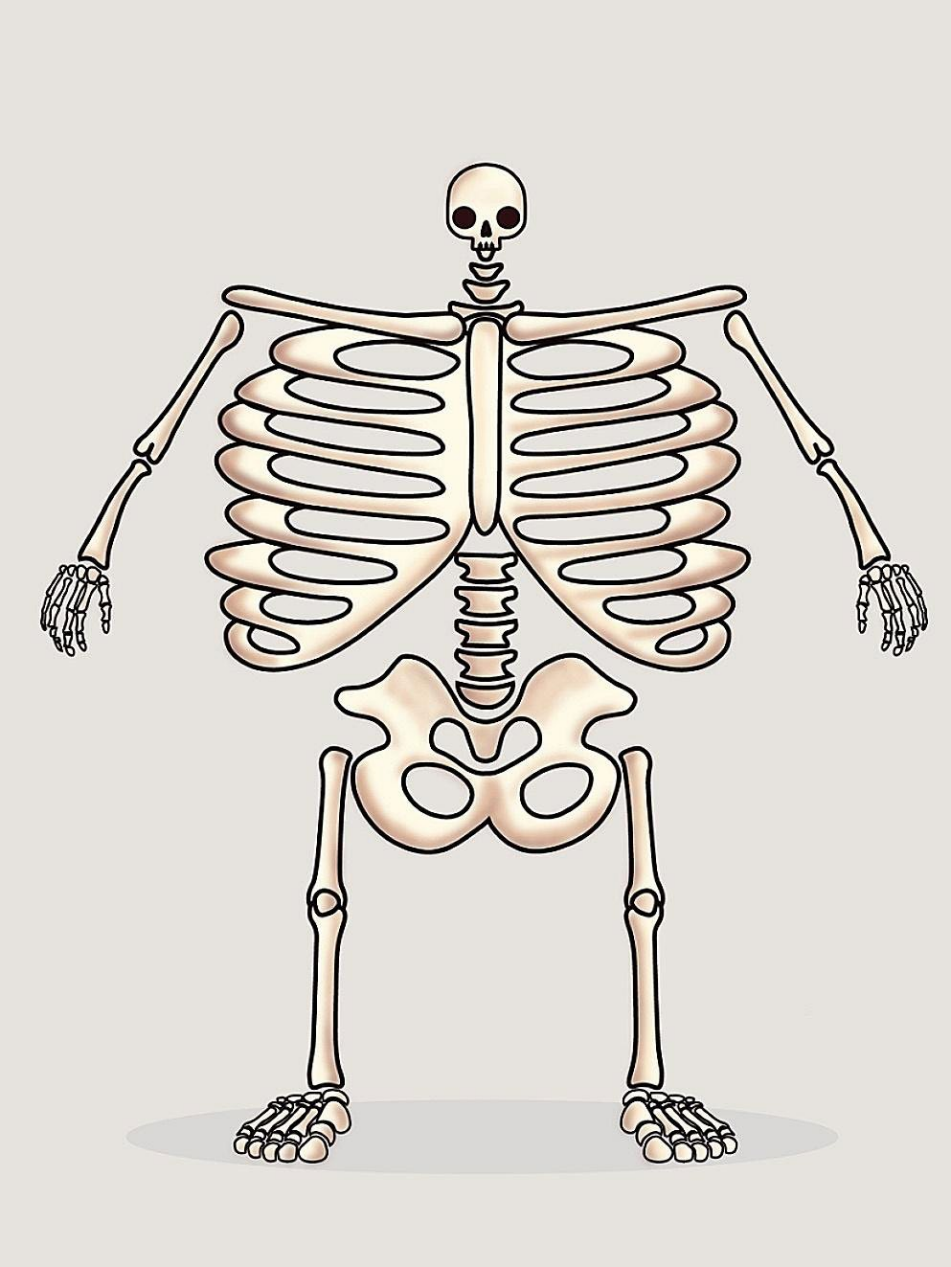
The [^18^F]PSMA-1007 UBUs homunculus. This figure visually represents [^18^F]PSMA-1007 UBUs’ distribution across the human skeleton, emphasizing high-incidence areas with scaled prominence to underscore their clinical significance

**Fig. 4:**
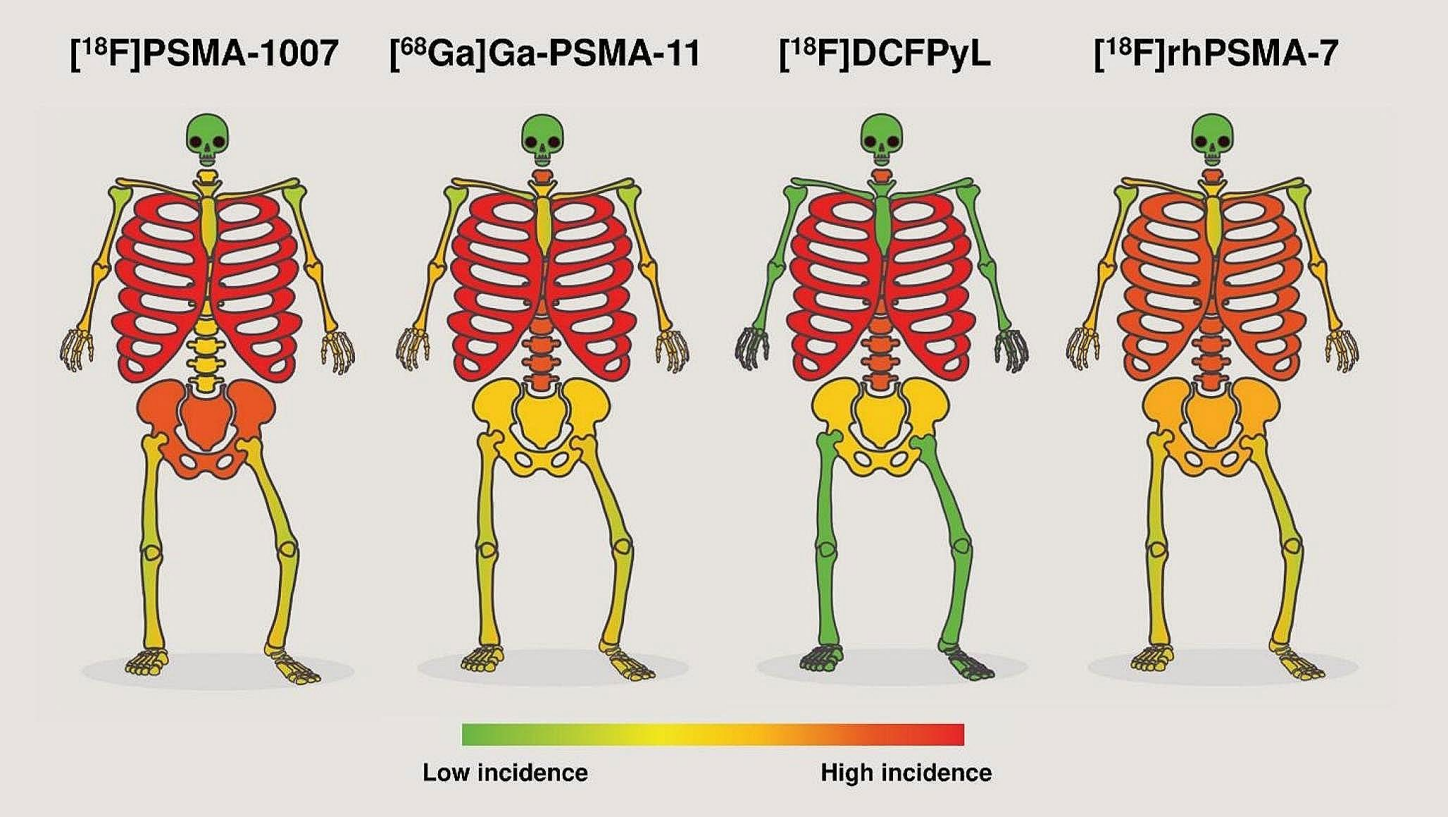
The distribution of UBUs according to the PSMA-targeted radiopharmaceutical used. This figure provides a visual representation of the distribution of UBUs throughout the human skeleton according to the PSMA-targeted radiopharmaceutical used, emphasizing high-incidence areas with hot colours to underscore their clinical significance

Regarding the anatomical localisation, most UBUs were reported in the ribs, spine and pelvic girdle, followed by the sternum, shoulder girdle and limbs [16–18, 20–30]. A less frequent UBUs location was the skull. In Fig. 3, we illustrate the topographical distribution of [^18^F]PSMA-1007 UBUs throughout the entire skeleton, drawing inspiration from Penfield’s human homunculus [31]. However, minor differences in UBU topography can be observed when comparing different PSMA-targeted tracers. A quantitative synthesis of the prominent UBU locations according to the PSMA-ligand used across the included studies is reported in Table 5 and visually represented in Fig. 4.

**Table 5:** Topographical distribution of UBUs according to the PSMA-ligand used across the included studies

UBUs site	Absolute number of reported UBUs (percentage)			
	**[**^**18**^**F]PSMA-1007** (no. pts: 1415)	**[**^**68**^**Ga]Ga-PSMA-11** (no. pts: 911)	**[**^**18**^**F]DCFPyL** (no. pts: 243)	**[18 F]rhPSMA-7** (no. pts: 160)
Total UBUs	743	229	88	120
Skull	3 (< 0.05%)	1 (< 0.5%)	/	/
Spine	90 (12%)	51 (23%)	28 (32%)	43 (36%)
Sternum	21 (3%)	2 (< 1%)	/	3 (2.5%)
Ribs	407 (55%)	124 (54%)	39 (44%)	45 (38%)
Shoulder girdle	18 (2%)	10 (4%)	/	4 (3.3%)
Pelvis girdle	177 (24%)	35 (15%)	21 (24%)	24 (20%)
Limbs	27 (4%)	6 (3%)	/	1 (< 1%)

Note: since some studies performed intra-individual comparisons by administering different radiopharmaceuticals to the same patients, the total number of subjects included in this analysis exceeds the number of patients reported in the results section of the included papers

When biopsied, UBUs were mainly related to benign conditions such as fibroblastic reaction, fibrous dysplasia, hyperplastic bone marrow, or Paget’s disease. Interestingly, some papers did not report any alteration in the biopsied bone marrow [17, 20, 22, 25–27, 30] (Table 4).

Several studies aimed to identify clinical risk factors associated with the occurrence of UBUs in PET scans. No relationship was observed between the frequency of UBUs and the clinical indication for the PET scan (primary staging vs. restaging). Additionally, serum PSA levels and Gleason Score were not considered risk factors for the appearance of UBUs [16, 20, 22, 27]. Ninatti et al. explored a potential correlation between the presence of UBUs and elevated white blood cell counts [28]. The same study also observed lower body mass index and bone density values (measured in Hounsfield Units), in patients presenting UBUs, though these findings were not statistically significant [28]. Given that focal uptakes in bones might not only represent false-positive findings but could also reflect the presence of bone metastases, numerous studies focused on the role of clinical, biochemical, and histopathological features in differentiating between benign and malignant causes of these uptakes. Within this context, PSA levels and PCa histology have emerged as potentially valuable indicators [20, 24, 26, 27, 30].

## Discussion

The present systematic review highlights the complexity of the UBUs phenomenon in PET/CT scans utilising PSMA-targeted ligands. Consistent with prior literature [29, 32], we identified [^18^F]PSMA-1007 as the tracer associated with a significantly higher rate of UBUs, especially in rib areas, across all examined settings. This topic is of increasing relevance, as the clinical use of [^18^F]PSMA-1007 is rising due to several reasons, including cyclotron-based production (which allows synthesising larger quantities of [^18^F]PSMA compared to [^68^Ga]Ga generators), the longer half-life, the lower positron range and the higher signal-to-background ratio [18, 33]. Despite [^18^F]PSMA-1007 being the tracer most frequently related to the presence of UBUs, renally excreted radiopharmaceuticals might also be associated with this phenomenon. Indeed, in the included studies, the percentage of patients with equivocal bony findings ranged from 0 to 23.9% [16, 18, 21, 24, 26, 29]. Similarly, in the OSPREY trial, which included a bone biopsy for 44 patients undergoing restaging for disease recurrence with [^18^F]DCFPyL, false positive findings were observed in about 15% of the patients included [34]. .

The precise pathophysiological mechanisms behind UBUs remain elusive. The initial hypothesis of free fluorine involvement has been challenged [35], with the chemical composition-driven affinity being partially responsible [36]. Nevertheless, PSMA radioligands with hydrophilic compositions also demonstrate false positive bone findings, suggesting this phenomenon is not unique to [^18^F]PSMA-1007 [29]. Furthermore, healthy bone marrow lacks PSMA immunohistochemical positivity, highlighting the limited understanding of these unspecific uptakes’ biological mechanisms [37]. However, at least in some instances, a morphological correlate seems likely, given that UBUs may persist in follow-up scans [22]; this calls for further research to elucidate their nature. As observed by the groups of Alberts [12] and Grünig [22], the technological shift towards digital PET devices introduces a further bias towards increased false positive findings. Notably, when comparing digital PET/MRI with analogue PET/CT scanners, the difference in false positive findings was not observed, perhaps due to the slightly reduced sensitivity of digital PET detectors in MRI scanners due to coils and the magnetic field [38]. The evolving technological landscape thus necessitates awareness of the trade-offs between sensitivity and specificity. Texture analysis of lesions emerges as a potential game-changer for differential diagnosis, although its practical application requires substantial datasets [39].

From a clinical perspective, the misinterpretation of UBUs can lead to inappropriate treatment decisions, intensifying the ongoing discussion about the stage migration phenomenon in cancer diagnosis and treatment [39–41]. In this regard, our systematic review focused on understanding the topography of UBUs by differentiating between PSMA-targeted ligands, thereby enhancing our knowledge base for image interpretation. Our primary finding is that the ribs are the most frequent site of UBUs, irrespective of the PSMA-targeted radiopharmaceutical used. Wang et al. [42] previously explored the distribution of bone metastases in a large cohort of PCa patients through bone scans, noting a predominant occurrence in the vertebrae and pelvis during the early stages [42]. Their study revealed that only 1% of patients exhibited bone metastases without involvement of the vertebrae and pelvis [42]. Given the differences in topography between UBUs and typical PCa bone metastatic patterns, we suggest that a single PSMA-avid focal uptake in the ribs is unlikely to be metastatic in most cases, regardless of the PSMA radiopharmaceutical used. In contrast, focal bone uptake involving the spine (the second most frequent UBU site for [^68^Ga]Ga-PSMA-11, [^18^F]DCFPyL, [^18^F]rhPSMA-7) or the pelvic girdle (the second most frequent site for [^18^F]PSMA-1007) presents more interpretive challenges. In these cases, analyzing imaging parameters may improve image interpretation. Indeed, some studies have focused on SUV_max_ [20, 24, 26, 27, 30], even with the goal of establishing a cutoff value for clinical decision-making [20]. Further research identifying clinical risk factors for bone metastases in patients with focal bone uptakes underscores the importance of integrating clinical context with imaging. This context includes factors such as PSA levels, histology [20, 24, 26, 27, 30] and even non-cancer-related parameters like white blood cell counts [28]. These insights suggest that nuclear medicine physicians must sometimes tailor their reports based on the radiopharmaceutical used, prioritizing pre-test metastatic probability over individual uptakes.

This systematic review acknowledges several limitations: (i) the heterogeneity of the included studies and the nature of the topic precluded data pooling and a meta-analysis. Merging prevalence rates from studies with varying inclusion criteria and different radiopharmaceuticals could lead to misleading outcomes; (ii) variability in reported data prevented a per-lesion analysis; (iii) our tracer-specific topographical assessment of UBUs is limited to data from only four PSMA-targeted tracers, due to the scarcity of comprehensive data on this topic in the current literature; (iv) most of the included studies were retrospective, and most patients did not undergo confirmatory biopsy of their UBUs, making it difficult to accurately determine the proportion of false positives versus true bone metastases. This limitation introduces a potential bias that should not be overlooked. Furthermore, although this review did not directly assess the preferential use of one radiopharmaceutical over another in clinical practice, it included a prospective study comparing [^18^F]PSMA-1007 and [^68^Ga]Ga-PSMA-11 [24], which suggests the equivalent performance of these tracers in nodal and distant metastasis staging. Additionally, a retrospective study by Seifert et al. [29] involving BCR patients indicated no significant difference in the detection rates of bone metastases between the two radiopharmaceuticals. This supports the premise that experienced physicians can effectively adjust for UBU findings, emphasizing the flexibility in the choice of PSMA-targeted agents in clinical practice. Finally, this review focused on radiopharmaceuticals labelled with positron emitters and excluded gamma-emitting tracers, such as [^99m^Tc]Tc-labeled PSMA radiopharmaceuticals, and PSMA-targeted radioligands used for therapeutic purposes, such as [^177^Lu]Lu-PSMA-617. The exclusion is due to the absence of reports in the available literature concerning UBUs observed in both diagnostic and post-treatment scintigraphic imaging.

## Conclusions

In conclusion, UBUs present a notable diagnostic challenge in a diverse range of patients undergoing PSMA PET scans for PCa, especially when using [^18^F]PSMA-1007. From our systematic review, we draw the following key conclusions:

- The ribs are the primary site of UBUs across all PSMA-targeted radiopharmaceuticals. Isolated rib uptakes are typically non-metastatic.
- Focal bone uptakes involving the spine or pelvic girdle, the second most frequent UBU sites for specific PSMA tracers, require careful interpretation due to their complexity.
- Evaluating imaging parameters, such as SUV_max_, in conjunction with clinical context assessment is crucial for accurately interpreting challenging cases.

## Electronic supplementary material

Below is the link to the electronic supplementary material.

Supplementary Material 1

## Data Availability

N.A.
